# Complete genome sequence of *Stackebrandtia nassauensis* type strain (LLR-40K-21^T^)

**DOI:** 10.4056/sigs.47643

**Published:** 2009-12-30

**Authors:** Chris Munk, Alla Lapidus, Alex Copeland, Marlen Jando, Shanmugam Mayilraj, Tijana Glavina Del Rio, Matt Nolan, Feng Chen, Susan Lucas, Hope Tice, Jan-Fang Cheng, Cliff Han, John C. Detter, David Bruce, Lynne Goodwin, Patrick Chain, Sam Pitluck, Markus Göker, Galina Ovchinikova, Amrita Pati, Natalia Ivanova, Konstantinos Mavromatis, Amy Chen, Krishna Palaniappan, Miriam Land, Loren Hauser, Yun-Juan Chang, Cynthia D. Jeffries, Jim Bristow, Jonathan A. Eisen, Victor Markowitz, Philip Hugenholtz, Nikos C. Kyrpides, Hans-Peter Klenk

**Affiliations:** 1 Los Alamos National Laboratory, Bioscience Division, Los Alamos, New Mexico, USA; 2 DOE Joint Genome Institute, Walnut Creek, California, USA; 3 DSMZ - German Collection of Microorganisms and Cell Cultures GmbH, Braunschweig, Germany; 4 Microbial Type Culture Collection, Institute of Microbial Technology, Chandigarh, India; 5 Biological Data Management and Technology Center, Lawrence Berkeley National Laboratory, Berkeley, California, USA; 6 Oak Ridge National Laboratory, Oak Ridge, Tennessee, USA; 7 University of California Davis Genome Center, Davis, California, USA

**Keywords:** aerobic, Gram-positive, non-acid-fast, mycelium producing, 2-hydroxy fatty acids-containing, *Glycomycetaceae*

## Abstract

*Stackebrandtia nassauensis* Labeda and Kroppenstedt (2005) is the type species of the genus *Stackebrandtia*, and a member of the actinobacterial family *Glycomycetaceae*. *Stackebrandtia* currently contains two species, which are differentiated from *Glycomyces* spp*.* by cellular fatty acid and menaquinone composition. Strain LLR-40K-21^T^ is Gram-positive, aerobic, and nonmotile, with a branched substrate mycelium and on some media an aerial mycelium. The strain was originally isolated from a soil sample collected from a road side in Nassau, Bahamas. Here we describe the features of this organism, together with the complete genome sequence and annotation. This is the first complete genome sequence of the actinobacterial suborder *Glycomycineae*. The 6,841,557 bp long single replicon genome with its 6487 protein-coding and 53 RNA genes is part of the *** G****enomic* *** E****ncyclopedia of* *** B****acteria and* *** A****rchaea * project.

## Introduction

Strain LLR-40K-21^T^ (=DSM 44728 = NRRL B-16338 = JCM 14905) is the type strain of *Stackebrandtia nassauensis*, which is the type species of the genus *Stackebrandtia* [1]. *S. nassauensis* was originally isolated by M. P. Lechevalier and subsequently described by Labeda and Kroppenstedt [1] during the course of a 16S rRNA survey of putative *Glycomyces* strains. The genus was named after Erko Stackebrandt, a German microbiologist of note, who has contributed significantly to the molecular systematics the *Actinobacteria*. At present the genus *Stackebrandtia* contains only one additional species: *S. albiflava,* isolated from a soil sample collected from a tropical rainforest in China [2] .Here we present a summary classification and a set of features for *S*. *nassauensis* strain LLR-40K-21^T^ together with the description of the complete genomic sequencing and annotation.

## Classification and features

A search of GenBank revealed no 16S rRNA reference sequences that were closely related to *S. nassauensis*. With 95% sequence similarity, the type strain *S. albiflava*, YIM 45751 [2], is the only cultivated strain with a sequence similarity above 91%, whereas a 16S rRNA gene sequence derived from a sample isolated from pig slurry (pig saw dust spent bedding in France, M982657, Snell-Castro *et al*., unpublished), represents the only related phylotype (with the same degree of sequence similarity as YIM 45751). Curiously, the type strains of the neighboring genus *Glycomyces* [3] were not within the 250 top hits in BLAST searches, with the 16S rRNA of type species *G. harbinensis* [3] sharing only 89% sequence similarity. Screening of environmental genomic samples and surveys reported at the NCBI BLAST server also showed no closely related phylotypes (with 93% sequence identity at the maximum), indicating a rather limited environmental occurrence of the species *S. nassauensis* (as of July 2009).

**Figure 1:**
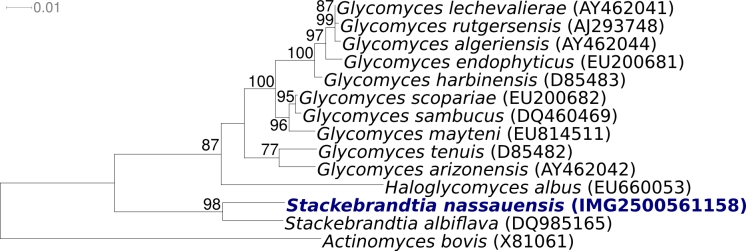
Phylogenetic tree of *S. nassauensis* strain LLR-40K-21^T^ and all type strains of the family *Glycomycetaceae*, inferred from 1,390 aligned characters [4] of the 16S rRNA sequence under the maximum likelihood criterion [5,6]. The tree was rooted with *Actinomyces bovis*, the type strain of the order *Actinomycetales*. The branches are scaled in terms of the expected number of substitutions per site. Numbers above branches are support values from 1,000 bootstrap replicates if larger than 60%. Lineages with type strain genome sequencing projects registered in GOLD [7] are shown in blue, published genomes in bold.

Figure 1 shows the phylogenetic neighborhood of *S*. *nassauensis* in a 16S rRNA based tree. The two 16S rRNA gene sequences in the genome of strain LLR-40K-21^T^ are identical and do not differ from the previously published 16S rRNA sequence generated from NRRL B-16338 (AY650268).

**Figure 2:**
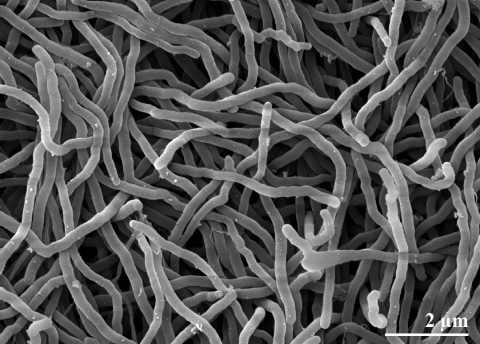
Scanning electron micrograph of *S. nassauensis* strain LLR-40K-21^T^ (Manfred Rohde, Helmholtz Centre for Infection Research, Braunschweig)

*S*. *nassauensis* strain LLR-40K-21^T^ cells are non-motile and filamentous, producing a with pale yellow to pale tan substrate mycelium on solid media [1] (Table 1 and Figure 2). Aerial mycelia are produced on some media and are white to yellowish-white in color [1]. Both aerial and substrate mycelia are approximately 0.5 µm in diameter [1]. Fragmentation of aerial or substrate mycelia into chlamydospores or zoospores has not been observed [1]. Cells stain Gram-positive, grow aerobically, and are non acid-fast [1]. Growth occurs at the temperature range of 15-37° C and in the presence of 4-9% NaCl. *S*. *nassauensis* LLR-40K-21^T^ is positive for hydrolysis or degradation of allantoin, casein, esculin, gelatin, hypoxanthine, starch and tyrosine but negative for adenine and xanthine [1]. The strain is capable of producing phosphatase and reducing nitrates; assimilation of acetate and malate is possible but not of benzoate, citrate, lactate, mucate, oxalate, propionate, succinate and tartarate [1]. Acid is produced aerobically from arabinose, cellobiose, dextrin, fructose, galactose, glucose, glycerol, lactose, maltose, mannose, melibiose, methyl α-D-glucoside, raffinose, rhamnose, salicin, sorbitol, sucrose, trehalose and xylose; but not from adonitol, dulcitol, erythritol, inositol, mannitol, melezitose or methyl-β-xyloside [1].

**Table 1:** Classification and general features of *S*. *nassauensis* strain LLR-40K-21^T^ according to the MIGS recommendations [8]

MIGS ID	Property	Term	Evidence code
	Current classification	Domain *Bacteria* Phylum *Actinobacteria* Class *Actinobacteria* Order *Actinomycetales* Suborder *Glycomycineae* Family *Glycomycetaceae* Genus *Stackebrandtia* Species *Stackebrandtia nassauensis* Type strain LLR-40K-21	TAS [9] TAS [10] TAS [11] TAS [11] TAS [11] TAS [1,11,12 TAS [1] TAS [1]
	Gram stain	positive	TAS [1]
	Cell shape	hyphae, aerial and substrate mycelium	TAS [1]
	Motility	non-motile	TAS [1]
	Sporulation	non-sporulating	TAS [1]
	Temperature range	mesophilic	TAS [1]
	Optimum temperature	15-37°C	TAS [1]
	Salinity	4-9g NaCl/l	TAS [1]
MIGS-22	Oxygen requirement	aerobic	TAS [1]
	Carbon source	glucose, maltose, mannose, cellobiose	TAS [1]
	Energy source	starch	TAS [1]
MIGS-6	Habitat	soil	TAS [1]
MIGS-15	Biotic relationship	free-living	NAS
MIGS-14	Pathogenicity	none	NAS
	Biosafety level	1	TAS [13]
	Isolation	road side soil	TAS [1]
MIGS-4	Geographic location	Nassau, Bahamas	TAS [1]
MIGS-5	Sample collection time	not reported	
MIGS-4.1 MIGS-4.2	Latitude / Longitude	25.066 / -77.339	NAS
MIGS-4.3	Depth	not reported	
MIGS-4.4	Altitude	not reported	

Evidence codes - IDA: Inferred from Direct Assay (first time in publication); TAS: Traceable Author Statement (i.e., a direct report exists in the literature); NAS: Non-traceable Author Statement (i.e., not directly observed for the living, isolated sample, but based on a generally accepted property for the species, or anecdotal evidence). These evidence codes are from the Gene Ontology project [14]. If the evidence code is IDA, then the property was observed for a living isolate by one of the authors or an expert mentioned in the acknowledgments.

### Chemotaxonomy

The murein of *S*. *nassauensis* strain LLR-40K-21^T^ contains *meso*-diaminopimelic acid as the diamino acid and N-glycolylmuramic acid as is found in members of the genus *Glycomyce*s. Ribose is the major cell wall sugar. Mannose has also been reported [1,2]. Reports about the presence of inositol, arabinose, xylose and glucose differ [1,2]. Galactose, which has been identified in all *Glycomyces* strains, has not been found in *Stackebrandtia* [1]. The fatty acid pattern of LLR-40K-21^T^ is dominated by saturated branched chain acids, anteiso-(ai-) C_17:0_ (26.8%), ai-C_15:0_ (2.8%), and iso-(i-) C_15:0_ (8.7%), i-C_16:1_ (2.1%), i-C_16:0_ (8.7%), i-C_17:0_ (9.0%). Unsaturated straight chain acids play only a limited role: C_17:1 cis9_ (1.8%), and C_16:1 cis9_ (3.1%). A significant amount of ai-C_17:0_ 2-OH (14.5%) and moderate amounts of hydroxylated fatty acids were also detected. Moderate amounts of saturated components including 10-methyl-branched heptadecanoic acid C_16:0_10 methyl (9.0%) and 10-methyl-C_17:0_ (1.4%) were also detected. The occurrence of 10-methyl branched heptadecanoic acid and i-branched 1-OH fatty acids is differential for *S.nassauensis* from members of the genus *Glycomyces* which lack these acids. Polar lipids identified are phosphatidylglycerol, diphosphatidylglycerol, like in members of the genus *Glycomyces*, and two additional yet unknown phospholipids are present. Phosphatidylinisitolmanosides (PIM) and phosphatidylglycerol (PI), which are present in the members of the genus *Glycomyces*, are absent; however, PI is present in *S. albiflava* [2]. Phosphatidylethanolamine (PE) and phosphatidylmethyl-ethanolamine (PME) were initially reported as absent in strain LLR-40K-21^T^ [1], but were later observed by Wang *et al.* [2]. The predominant menaquinones are MK-10 (H_4_), MK-10 (H_6_), MK-11 (H_4_) and MK-11 (H_6_), different from the patterns observed from the members of the genus *Glycomyces* which contain menaquinones with 9-12 isoprene units with various degrees of hydrogenation [1]. Mycolic acids are absent [1].

## Genome sequencing and annotation

### Genome project history

This organism was selected for sequencing on the basis of its phylogenetic position, and is part of the *** G****enomic* *** E****ncyclopedia of* *** B****acteria and* *** A****rchaea * project. The genome project is deposited in the Genomes OnLine Database [7] and the complete genome sequence in GenBank. Sequencing, finishing and annotation were performed by the DOE Joint Genome Institute (JGI). A summary of the project information is shown in Table 2.

**Table 2:** Genome sequencing project information

MIGS ID	Property	Term
MIGS-31	Finishing quality	Finished
MIGS-28	Libraries used	Two genomic libraries: 8kb pMCL200 and fosmid pcc1Fos Sanger libraries. One 454 pyrosequence standard library
MIGS-29	Sequencing platforms	ABI3730, 454 GS FLX
MIGS-31.2	Sequencing coverage	11.0× Sanger; 29× pyrosequence
MIGS-30	Assemblers	Newbler version 1.1.02.15, phrap
MIGS-32	Gene calling method	Prodigal 1.4, GenePRIMP
	INSDC ID	CP001778
	Genbank Date of Release	not yet
	GOLD ID	Gc01107
	NCBI project ID	19713
	Database: IMG-GEBA	2501939631
MIGS-13	Source material identifier	DSM 44728
	Project relevance	Tree of Life, GEBA

### Growth conditions and DNA isolation

*S. nassauensis* strain LLR-40K-21^T^, DSM 44728, was grown in DSMZ medium 553 (GPHF Medium) [15] at 28°C. DNA was isolated from 1-1.5 g of cell paste using Qiagen Genomic 500 DNA Kit (Qiagen, Hilden, Germany) with lysis modification DALM according to Wu *et al*. [16].

### Genome sequencing and assembly

The genome was sequenced using a combination of Sanger and 454 sequencing platforms. All general aspects of library construction and sequencing can be found at the JGI website. 454 Pyrosequencing reads were assembled using the Newbler assembler version 1.1.02.15 (Roche). Large Newbler contigs were broken into 7,157 overlapping fragments of 1,000 bp and entered into assembly as pseudo-reads. The sequences were assigned quality scores based on Newbler consensus q-scores with modifications to account for overlap redundancy and to adjust inflated q-scores. A hybrid 454/Sanger assembly was made using the phrap assembler (High Performance Software, LLC). Possible mis-assemblies were corrected with Dupfinisher or transposon bombing of bridging clones [17]. Gaps between contigs were closed by editing in Consed, custom primer walk or PCR amplification. A total of 308 Sanger finishing reads were produced to close gaps and to raise the quality of the finished sequence. The error rate of the completed genome sequence is less than 1 in 100,000. The final assembly consists of 81,931 Sanger and 851,638 pyrosequence reads. Together all sequence types provided 40.0× coverage of the genome.

### Genome annotation

Genes were identified using Prodigal [18] as part of the Oak Ridge National Laboratory genome annotation pipeline, followed by a round of manual curation using the JGI GenePRIMP pipeline [19]. The predicted CDSs were translated and used to search the National Center for Biotechnology Information (NCBI) nonredundant database, UniProt, TIGRFam, Pfam, PRIAM, KEGG, COG, and InterPro databases. Additional gene prediction analysis and manual functional annotation was performed within the Integrated Microbial Genomes Expert Review (IMG-ER) platform [20].

## Genome properties

**Figure 3:**
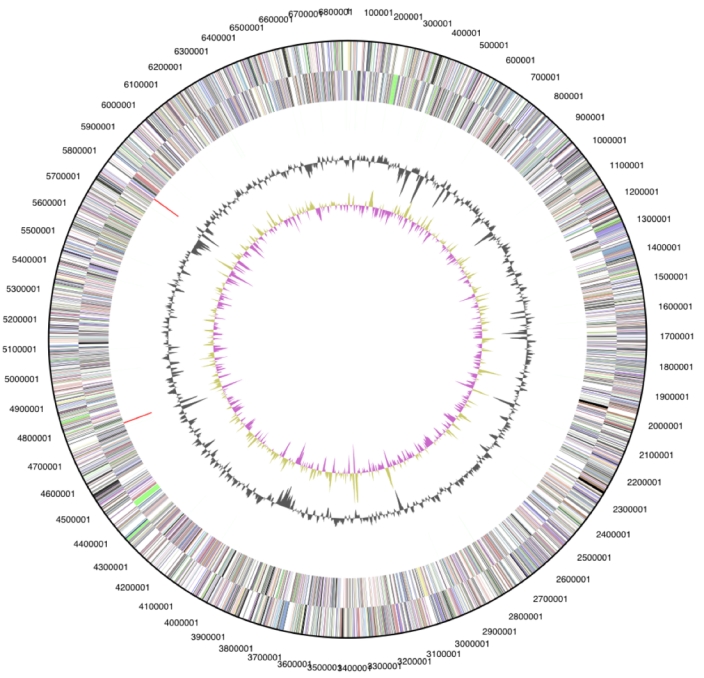
Graphical circular map of the genome. From outside to the center: Genes on forward strand (color by COG categories), Genes on reverse strand (color by COG categories), RNA genes (tRNAs green, rRNAs red, other RNAs black), GC content, GC skew.

The genome is 6,841,557 bp long and comprises one circular chromosome with a 68.1% GC content (Table 3 and Figure 3). Of the 6,450 genes predicted, 6,487 were protein coding genes, and 53 RNAs; One hundred eight pseudogenes were also identified. The majority of the protein-coding genes (66.8%) were assigned a putative function while those remaining were annotated as hypothetical proteins. The properties and the statistics of the genome are summarized in Table 3. The distribution of genes into COGs functional categories is presented in Table 4.

**Table 3:** Genome Statistics

Attribute	Value	% of Total
Genome size (bp)	6,841,557	100.00%
DNA Coding region (bp)	6,296,517	92.03%
DNA G+C content (bp)	4,661,422	68.13%
Number of replicons	1	
Extrachromosomal elements	0	
Total genes	6,540	
RNA genes	53	0.81%
rRNA operons	2	
Protein-coding genes	6,487	99.20%
Pseudo genes	108	1.65%
Genes with function prediction	4,368	66.79%
Genes in paralog clusters	1,454	22.23%
Genes assigned to COGs	4,215	64.45%
Genes assigned Pfam domains	4,474	68.41%
Genes with signal peptides	1,698	25.96%
Genes with transmembrane helices	1,858	28.41%
CRISPR repeats	4	

**Table 4:** Number of genes associated with the general COG functional categories

Code	value	%age	Description
J	197	4.1	Translation, ribosomal structure and biogenesis
A	2	0.0	RNA processing and modification
K	653	13.5	Transcription
L	184	3.8	Replication, recombination and repair
D	31	0.6	Cell cycle control, mitosis and meiosis
Y	0	0.0	Nuclear structure
V	126	2.6	Defense mechanisms
T	348	7.2	Signal transduction mechanisms
M	214	4.4	Cell wall/membrane biogenesis
N	2	0.0	Cell motility
Z	1	0.0	Cytoskeleton
W	0	0.0	Extracellular structures
U	38	0.8	Intracellular trafficking and secretion
O	151	3.1	Posttranslational modification, protein turnover, chaperones
C	275	5.7	Energy production and conversion
G	436	9.0	Carbohydrate transport and metabolism
E	367	7.6	Amino acid transport and metabolism
F	102	2.1	Nucleotide transport and metabolism
H	229	4.7	Coenzyme transport and metabolism
I	178	3.7	Lipid transport and metabolism
P	212	4.4	Inorganic ion transport and metabolism
Q	169	3.5	Secondary metabolites biosynthesis, transport and catabolism
R	622	12.9	General function prediction only
S	304	6.3	Function unknown
-	2325	35.6	Not in COGs
